# Supplementary material for: Are carbon emissions trading and green financial instruments synergistic? -Comprehensive quantitative research based on content analysis
Source: PLoS One. 2024 Mar 7;19(3):e0298601. doi: 10.1371/journal.pone.0298601 (PMC10919601; doi:10.1371/journal.pone.0298601)
Supplement: S1 Data — (ZIP) [file pone.0298601.s001.zip › Data/地级市Z_F工作报告.zip!/╔╜╢1⁄2/╝├─■╩╨/└┤╫╘╣1⁄2╓┌║┼í╛┬φ┐╦╩2╛▌═°í┐_2013.docx]

各位代表：

　　现在，我代表济宁市人民政府向大会报告工作，请予审议，并请市政协委员和其他列席会议的同志提出意见。

　　一、2012年工作回顾

　　过去的一年，面对复杂严峻的国内外经济形势，全市上下在中共济宁市委的坚强领导下，深入落实市第十二次党代会精神，牢牢把握科学发展跨越发展总基调，

紧盯“三个高于、三个提高”任务目标，解放思想，开拓创新，攻坚克难，拼搏实干，各项工作取得新成绩，新一届政府实现良好开局。

　　———经济实现平稳较快发展。预计全市地区生产总值达到3210亿元、增长11%；公共财政预算收入完成245.6亿元、增长18.6%；固定资产投资1740亿元、增长22%；社会消费品零售总额1300亿元、增长15%，居民消费价格同比上涨2.1%。金融机构年末存款余额3090亿元、贷款余额1970亿元，分别比年初增加460亿元、320亿元。

　　———产业结构得到优化提升。全市规模以上工业企业发展到1450家，四大千亿级产业规模实力不断增强，拉动工业增加值增幅8个百分点。战略性新兴产业发展迅速，实现销售收入540亿元、增长47%。高新技术产业产值占工业比重提高到20.6%。服务业实现增加值1150亿元、占比提高1个百分点。

　　———发展动力和活力显著增强。国有资产管理体制改革纵深推进。投融资体系建设取得新进展，市产业发展投资公司、融资担保公司与城建投资公司形成“三驾马车”，引进民生银行、莱商银行，5家村镇银行和5家保险公司开业，全市股权投资基金达到15支，小额贷款公司发展到19家。全年取得重要科技成果150项、专利授权5000项，济宁高新区产学研基地建成使用，10名国家“千人计划”专家来我市创新创业。对外开放进一步扩大，新批外资项目49个，实际利用外资7.7亿美元；新增境外投资企业28家，完成投资5.4亿美元。全民创业行动扎实开展，新增民营企业3003家，民营经济增加值增长12.5%。

　　———城乡面貌发生明显变化。中心城区实施重点项目96个、完成投资121.9亿元，一批公共基础设施建成使用，升级改造主干道和背街小巷25条，改造棚户区58.9万平方米，新增绿地164.9万平方米、环保公交车200辆、天然气用户1.5万户、供热面积310.6万平方米。农村环境综合整治成效明显，完成3640个村整治任务，硬化道路7670公里，建成垃圾中转站278座，配备保洁员1.4万人，农村环境得到净化美化，城乡环卫一体化长效机制初步形成。

　　———生态环境质量持续提升。节能减排任务全面完成，单位生产总值能耗、主要污染物排放继续下降。水污染治理力度加大，主要河流水质稳定达标。大气污染防控成效明显，空气质量改善幅度居全省前列。新增造林面积19万亩，建成国家级森林公园和湿地公园2处。18个乡镇、34个村成为国家级生态镇村。

　　———民生保障水平不断提高。城镇居民人均可支配收入和农民人均纯收入分别达到25500元、10020元，增长13.8%和15%。新增城镇就业12.7万人，农村劳动力转移就业11.9万人。城乡养老、医保制度实现全覆盖，保障水平不断提高。文化惠民工程深入实施，城乡居民精神文化生活更加丰富。为民承诺所办10件实事如期兑现。

　　一年来，重点做了以下工作：

　　（一）全力突破工业经济。实施“工业强市”战略，开展“为企业排忧解难服务”活动，强化监测预警，落实要素保障，实现了工业经济企稳回升。围绕做大做强主导产业骨干企业，制定出台扶持千百亿工业企业发展政策，推进千户企业改造提升、利税过千万元企业信息化改造、骨干企业创百亿“三大工程”。实施了100个重大技改项目，完成投资560亿元、增长26%。太阳纸业、华勤集团销售收入突破300亿元，润峰集团、翔宇化纤、荣信化工突破100亿元。积极推进企业技术创新，新建重点实验室和院士工作站15个，国家煤及煤化工检测中心、鲁南技术产权交易中心正式运营，14个高水平研发团队入驻鲁南工程技术研究院。着眼培育壮大中小企业群体，实施金融助推千家中小企业发展计划，新增中小微企业贷款160亿元。

　　（二）集中攻坚重大项目。深入开展“大项目突破年”活动，在北京、上海、香港等地区开展了系列招商活动，加强与世界500强、大型央企和知名民企的对接，引进了一批重大产业项目，到位市外国内资金671亿元、增长42%。深度推进与省直部门、金融机构和驻济大企业战略合作，签订合作协议23项。坚持每季度举行一次重大项目集中开工活动，建立领导包保、分类管理、部门联系推进制度，优化发展环境，千方百计保项目落地建设。全年累计新开工亿元以上项目328个、同比增加147个，娃哈哈灌装饮料、吉利自动变速箱等一批产业项目竣工投产。

　　（三）夯实“三农”工作基础。认真落实强农惠农富农政策，发放政策性补贴9.6亿元。粮食生产实现"九连增"，总产达到113亿斤。推进农民土地承包经营权流转，标准化种养基地发展到381万亩，新增农业产业化龙头企业56家、农民专业合作社854家，农产品质量安全示范区发展到140万亩。农业综合机械化水平达到85%。南水北调干线主体工程和治淮东调南下续建工程竣工。加强农田水利基本建设，解决了37.4万农村居民饮水安全问题，12个县市区纳入全国小型农田水利重点县建设范围。

　　（四）强力推动县域发展。实施“县域经济倍增计划”，加大产业布局、转移支付、对口帮扶、人才培训等扶持力度，市财政一般性转移支付新增2.5亿元，落实帮扶资金4.7亿元、项目184个。县域公共财政预算收入增幅高于全市平均2个百分点。赋予14个省级开发区完全县级管理权限，10个开发区突破百亿元，园区固定资产投资、提供税收分别占县域的59%、42%。建立向乡镇倾斜的政策导向机制，实行市直部门联系包保乡镇制度，全市公共财政预算收入增幅超过30%的乡镇发展到108个。

　　（五）加速推进城镇化进程。以开展“城市建设管理年”活动为抓手，持续实施城镇化追赶战略，全市城镇化率提高2个百分点、达到46%。出台支持北湖新区加快发展政策，从管理职能和保障措施等多方面构建起完备的支撑体系。大力推进省运会场馆建设，奥体中心体育场试运行，指挥中心主体竣工。济宁高新区以廖沟河景观打造和系列科技场馆建成为标志，布局框架进一步拉开。济北新区任城行政中心正式启用，金宇路升级改造加快（上接1版）金宇路升级改造加快实施。“三河六岸”开发和老城区东南片区改造全面启动，万达广场等城市综合体项目加紧建设。北湖连通桥、南二环京杭运河特大桥建成，临菏路济宁段全线贯通。太白楼路东西延伸线、济徐高速东平至济宁段竣工通车。滨湖大道济宁至鱼台段路基和桥梁主体施工基本完成。济宁曲阜机场新开通5条航线。梁济运河济宁城区段下游11公里疏浚工程全面完成。金乡、微山、梁山、曲阜等县市老城改造和新城开发建设力度加大、特色凸显，小城镇建设步伐加快。进一步理顺市区城建管理体制，构建起市控区统、条块互动、运转高效的城市管理格局。

　　（六）加快建设文化强市。大力实施文化建设“突破曲阜”战略，出台一揽子扶持政策，支持曲阜文化建设率先突破。曲阜片区大遗址规划建设扎实推进，国家级文化产业示范园区加紧实施。尼山圣境、东方文博城等一批项目开工建设，兴隆文化园、水浒文化主题公园等项目加快推进，微山湖景区被批准为省级旅游度假区。成功举办第二十九届国际孔子文化节等系列文化活动。市县国有文艺院团改革全面完成，济宁报业传媒集团、演艺集团挂牌成立。全市镇街全部建成综合文化站，95%以上的村居建成文化大院、农家书屋。

　　（七）加强民生和社会事业建设。在全省率先启动民生保障十大体系建设。大力实施教育优先战略，新改扩建公办幼儿园462所，改造中小学校舍52万平方米，省级规范化学校达到111所。实施学生营养改善计划和中小学健康饮水工程，向困难家庭学生发放营养补助金4500万元。出台支持高校建设发展意见，促进地校融合发展。医改五项重点任务圆满完成，“先看病后付费”就诊模式全面推开，城镇职工和居民医疗保险实现门诊统筹。开展“百名医疗专家进千村”、“万名保健医生进农户”活动，有效缓解了群众看病难问题。新型社会救助体系基本形成，困难群众、特殊群体、优抚对象生活得到有效保障。“四位一体”养老经验在全省推广。建设保障性住房1.38万套，完成农村危房改造1.35万户。人口自然增长率控制在7‰以内。省运会筹备工作扎实推进。加强和改进食品安全监管，建成放心粮油食品示范店116家。深化“平安村居、社区、单位”创建活动，开展打霸除恶专项行动，群众安全感普遍增强。实行领导干部公开接访、接听市长公开电话制度，重点信访问题和社会矛盾得到及时化解。安全生产形势持续平稳，应急管理工作不断加强，社会保持和谐稳定。援疆工作积极推进。国防动员、民兵预备役和双拥共建工作富有成效。国家安全、新闻出版、广播影视、民族宗教、公共资源交易、住房公积金、外事侨务、统计、物价工作取得新成绩。档案史志、人防地震、水文气象、妇女儿童、老龄、残疾人、慈善、红十字等项事业加快发展。

　　一年来，我们致力于打造法治政府、服务型政府和廉洁政府，不断强化政府自身建设。主动接受人大依法监督、政协民主监督，人大代表建议、政协提案办复率均为100%。深入实施依法行政“五五”规划和“六五”普法，行政复议改革试点经验在全国推广。开展“行政程序年”活动，重大行政决策更加科学规范、公开透明。全面落实党风廉政建设责任制，扎实开展“双评”和纠风专项治理，加强审计监督，严肃查办了一批腐败案件。

　　各位代表，过去的一年，在形势十分复杂、任务空前繁重的背景下，我市经济社会发展取得这样的好成绩实属不易。这得益于省委、省政府和市委的正确领导，离不开市人大、市政协的监督支持，凝结着全市广大干部群众的心血汗水。全市上下特别是广大基层的同志面对困难不退缩，殚精竭虑谋发展，扑下身子抓落实，表现出苦干实干、勇于担当、无私奉献的“精气神”。在此，我代表市人民政府，向全市工人、农民、知识分子和干部，向各级人大代表、政协委员，各民主党派、工商联、人民团体和各界人士，向中国人民解放军驻济部队指战员、武警官兵和公安干警，向中央、省驻济单位，向来济宁兴业发展的投资者和建设者，向关心支持济宁现代化建设的港澳台同胞、海外侨胞和国际友人，表示衷心感谢和崇高敬意！

　　我们也清醒地认识到，全市发展中还面临许多矛盾和问题。主要是：人均经济规模和财力偏低；经济结构性矛盾仍然突出，县域发展不平衡；自主创新能力不足，节能减排压力大；城市管理、公共服务和社会管理创新亟待加强；政府效能建设仍需进一步强化。对此，我们将高度重视，采取有力措施加以解决。

　　二、2013年重点工作

　　今年是深入贯彻落实党的十八大精神的开局之年，是实施“十二五”规划承前启后的关键一年，也是市委、市政府新一轮重大战略部署的落实年、突破年。经济社会发展既面临着严峻挑战，更面临着难得机遇。随着我市一系列重大决策部署的深入实施，一批重大产业项目的竣工投产，一些关系济宁长远发展制约因素的有效破解，支撑全市跨越发展的条件更加稳固。只要我们保持奋发有为的精神状态，坚定攻坚克难的必胜信念，团结拼搏，开拓进取，就一定能够乘势而上，不断开创科学发展跨越发展新局面！

　　今年的政府工作，要以“落实年、突破年”为总要求，深入学习贯彻党的十八大精神，坚持以邓小平理论、“三个代表”重要思想、科学发展观为指导，紧紧围绕主题主线，以提高经济增长质量和效益为中心，把握“稳定增势、提速转型、争先进位”工作基调，进一步深化改革开放，强化创新驱动，注重统筹发展，突出工业化、城镇化“两化并进”，以项目建设为引领，集中突破工业发展、突破城市建设、突破县域经济、突破文化建设、突破生态建设，加快构建民生保障体系，实现经济持续快速健康发展和社会和谐稳定。主要预期目标安排为：地区生产总值增长11.5%，公共财政预算收入增长16%，规模以上工业增加值增长14%，固定资产投资增长23%，社会消费品零售总额增长15%以上，外贸进出口增长9%，实际利用外资稳步增加，城镇居民人均可支配收入和农民人均纯收入均增长12%，城镇登记失业率控制在3%以内，居民消费价格涨幅控制在3.5%左右，全面完成省下达的节能减排约束性目标，人口自然增长率控制在7‰以内。

　　完成上述目标任务，需要我们着眼全局、科学谋划，突出重点、统筹兼顾，在工作推进中体现“六个更加注重”的基本要求。一是更加注重加快发展。牢牢扭住经济建设这个中心，一心一意谋发展搞建设，力争主要经济指标增幅高于全省、高于周边、高于上年，努力提升全市经济总量和人均水平。二是更加注重转型发展。以高端高质高效发展为方向，加快经济战略转型，构建现代产业体系，促进工业化、信息化、城镇化、农业现代化同步发展。三是更加注重创新发展。坚持创新驱动、人才引领，引导和支持企业通过创新增强核心竞争力，激发经济增长的活力和动力。四是更加注重统筹发展。力促强县率先发展、中等县加速崛起、薄弱县跨越赶超；积极推动城乡发展一体化，加快建立完善的基础设施和公共服务体系。五是更加注重生态发展。强化资源节约集约利用，强力推进节能减排，大力发展循环经济，加快建设资源节约型、环境友好型社会。六是更加注重和谐发展。坚持以人为本，多谋民生之利，多解民生之忧，持续加大民生投入，加快构建民生保障体系，让广大人民群众生活得更加幸福！

　　重点抓好十个方面的工作。

　　（一）以做大做强工业经济为引擎，加快推进经济战略转型。工业是强市之基。把工业作为做强实体经济基础、调整优化产业结构的重要抓手，实施“1351”十大产业百千亿培植工程，努力提高经济发展的质量和效益。

　　加快新型工业化进程。一是推动传统产业转型升级。通过规划引领、项目带动，推进煤化工、装备制造、食品工业、纺织服装等产业迅速膨胀规模，尽快形成千亿级产业。突出抓好100项省级重点技改项目建设，技改投资超过700亿元、占比达到60%以上。落实骨干企业扶持、中央省属驻济企业加快发展政策，“一企一策”为企业排忧解难，以龙头企业的发展壮大，带动重点产业加速提升，年内新增百亿元企业2家。二是培育发展战略性新兴产业。完善产业规划布局，制定专项实施方案，重点延伸新能源、LED、电动汽车、生物医药、节能环保等产业链条，抓好高端装备制造、光电信息、新材料产业基地建设。三是加快信息化和工业化融合。实施“信息化行动计划”，培育100家利税过千万元信息化示范企业，建设100个重点示范应用项目。推动信息网络技术广泛应用，努力建设“智慧济宁”、“数字矿山”。四是扶持发展中小微企业。实施中小企业“百家成长千家培育工程”，在融资担保、政府采购、人才培训等方面提供政策帮扶。持续掀起全民创业热潮，大力发展民营经济，打造一批高素质企业家队伍，催生一批小而专、小而精、小而强的民营企业群体，形成梯次成长、集群发展格局。

　　加快园区转型升级。按照产业分区定位，推动各类生产要素向园区集中，以大企业为龙头、主导产品为核心，带动一批功能互补、配套协作的中小企业快速成长，形成特色突出、层级分明的产业集群。进一步完善园区基础设施，强化金融、研发、信息等服务平台建设，在更高层次上为入园企业提供增值服务。加快千亿级园区创建步伐，支持邹西大工业板块加速崛起，推进兖州工业园、经济开发区联合争创国家级开发区，年内12个省级园区突破百亿级，2个突破500亿元。

　　全力推进大项目建设。坚持以产业大项目带动工业突破，集中抓好长三角、珠三角等重点地区和日韩、欧美等发达国家的招商，加强与世界500强、央企的联络对接，跟踪落实签约项目和战略合作协议，创新高层推动和产业招商、企业招商、园区招商方式，鼓励骨干企业拿出优势资源与国内外大公司合资合作，力争在大项目引进上取得突破。优化投资环境，减少行政审批程序和行政事业性收费，开辟“绿色通道”，集中力量抓调度、抓推进，确保项目顺利落地建设、按时投产达产。年内每个县市区至少新上20个亿元以上产业项目、2个过10亿元工业项目。

　　（二）以提高综合产出效益为着力点，加快发展现代农业。坚持工业反哺农业、城市支持农村和多予少取放活方针，引导更多的资本、技术、人才等先进生产要素向“三农”领域倾斜，促进农业增效、农民增收、农村经济更好更快发展。一是调整优化农业结构。抓好现代农业粮食产业项目，开展高产创建活动，力争粮食生产“十连增”。发展壮大大蒜、食用菌、苗木花卉等高价值经济作物，实施渔业转型升级“六大工程”，扩大畜牧业生态健康养殖规模。有序推进农民土地承包经营权流转，扶持优质农产品连片规模种植。因地制宜发展设施农业、观光农业、生态农业和都市农业。二是提升产业化经营水平。实施农产品质量提升工程，推进农业标准化生产，完善基层农技服务体系，发展新型农村合作组织，搞好良种繁育、引进和推广，扩大农产品质量安全示范区规模，抓好品牌创建，提升济宁农产品的知名度、竞争力和经济效益。加强农业龙头企业培植，促使现有骨干企业增量提质，鼓励各类投资者进入农业产业化领域，创建基地型、链条式龙头企业。年内新增农业产业化龙头企业50家、农产品质量安全示范区50处、“三品一标”认证50个。三是强化农业基础支撑。加大农业科技投入，加强对基层技术人员和生产大户的科技培训，提升农业物质技术装备水平。加快推进现代水利示范市建设，构建完备的水资源调配网络，完成12个全国小型农田水利重点县年度建设任务。抓好农业综合开发和扶贫开发，提高农业综合生产能力。

　　（三）以载体建设为支撑，提速发展现代服务业。把发展服务业作为转方式调结构的突破口，推进服务业重点项目建设，力争服务业占比提高2个百分点。加快发展金融业，搞好金融招商，引进光大银行，加强与平安集团、国信证券等金融机构的合作，支持济宁银行跨区域经营，引导金融机构到县乡设立营业网点，用好债券、基金、信托等金融工具，助推实体经济发展。紧跟先进制造业发展步伐，推进运河物流交易服务中心、铁水联运等项目建设，大力发展内河集装箱运输和临港物流产业。按照社会化服务、专业化分工要求，加快工业企业二、三产业分离，推动研发、设计等业务外包。集中突破文化旅游业，提速推进尼山圣境、兴隆文化园等重大项目，汶上宝相宫景区建成开放，支持微山湖、水泊梁山创建5A级景区。加强“孔孟之乡、运河之都、文化济宁”城市形象品牌宣传，策划推出一批文化旅游演艺活动。组建孔孟旅游集团，整合旅游资源，规划设计精品线路，与国内知名旅行社、景区景点、周边城市实现有机对接、客源共享。科学布局城乡商贸网点，搞好大型商贸批发市场与城市建设的衔接，形成一批特色商业街区、专业市场、购物广场，解决“有城无市”的问题，努力把济宁建成鲁西南地区重要的商贸中心。

　　（四）以重大工程为牵动，加快推进城镇化进程。更大力度实施城镇化追赶战略，年内城镇化率提高3个百分点以上。

　　强力突破中心城区。一是坚定不移推进北湖新区建设。高起点做好各类专项规划和单体设计，完善道路管网、水电气暖等基础设施配套。加快省运会系列场馆建设，指挥中心、媒体中心、“一场三馆”、水上运动基地、飞碟靶场等场馆竣工交付使用。二是彰显高新区科技新城魅力。加快大学科技园、创意及工业设计园等项目建设，建成科技中心“一体六馆”，推进印象吟龙湾和廖沟河湿地工程建设，密切新城开发与产业发展的有机衔接，促进高新区向国际化科技新城迈进。三是提升济北新区城市功能。以任城行政中心北迁为带动，深度拓展东西两翼商务区建设，完成金宇路升级改造、北二环建设任务。四是加快老城区重点片区建设。提速推进万达广场等城市综合体和东南片区开发，完善城区雨污分流等配套基础设施，启动华能黄屯热电厂建设，新增供热面积100万平方米、天然气用户1万户。完成城区所有背街小巷改造任务。高水平实施“三河六岸”综合开发，突出抓好大运河文化产业带连片建设，开工一批地标性建筑和独特景观，拉开西部城区布局框架，展现城河相映、生态宜人的城市风貌。

　　加速组群结构城市融合发展。建立都市区融合发展协调推进机制，做好各板块间行动规划和制度设计，在基础设施、信息网络、产业布局、公交一体化等方面取得实质性突破。依托崇文大道等城际路网，科学规划布局重点产业园区，引导相关县市区集中实施一批先导性大项目，打造特色产业集聚带。积极争取都市区行政区划调整，完成核心区乡改镇、镇改街道工作。加快都市区大公交体系建设，逐步开通中心城区至县域“零换乘”公交，推进曲阜高铁站连接线工程早日竣工，打造组群结构城市“半小时”都市圈。加速济徐高速济宁段、东外环南延、滨河大道济宁至梁山段建设，滨湖路二期济宁至鱼台段竣工通车，提高城际交通通达能力。

　　统筹抓好县城和小城镇建设。坚持老城改造与新区拓展齐抓，功能提升与产业发展并举，增强县域城市承载能力和服务功能。抓住全省开展“百镇建设示范行动”机遇，分层次推进小城镇建设，以10个省市级示范镇为重点，加大城镇基础设施配套，完善文化、教育等公共服务功能，促进人口向小城镇集聚。尊重农民意愿，稳妥实施新型农村社区建设。深化户籍制度改革，有序推进农业转移人口市民化。

　　提升城市经营管理水平。放开搞活城市开发建设市场，充分运用市场机制，走出一条依靠市场力量开发建设城市的新路径。完善管理体制，创新运行机制，强化街道、社区在城市管理中的基础地位。发挥好“数字化”城管平台作用，全面推行市场化、网格化、精细化管理。深入实施畅通工程，努力解决行车难、停车难问题。

　　（五）以激发内生动力为突破口，促进县域经济跨越赶超。纵深推进“县域经济倍增计划”，严格落实政策，完善帮扶机制，挖掘发展潜力，推进县域均衡协调发展。

　　加大县域发展扶持力度。积极争取上级政策支持，千方百计强化要素保障，在项目、资金、土地、人才等方面落实具体措施。加强基础设施建设，在交通设施、水利工程和城市公用设施等方面给予更多倾斜。对薄弱县加大财政转移支付、税费返还力度。督促对口帮扶县市区、部门单位和企业落实责任，协力推动县域经济实力明显提升，确保地区生产总值、财政收入、固定资产投资增幅高于全省平均水平，在全国、全省县域排名位次前移。

　　培植壮大特色主导产业。立足资源优势和产业基础，推进县域经济特色化、差异化、集群化发展，加快培植一批重点行业、特色产业。在农产品精深加工、工业配套协作、文化旅游资源开发等领域布局特色项目，延伸产业链条，增强竞争优势。年内各县市区新增规模以上企业20个。

　　夯实镇域经济基础。牢固树立“产业兴镇、商贸活镇”理念，加快建设一批独具特色的工业重镇、商贸强镇和旅游名镇。支持乡镇招商项目集聚园区发展，持续增强镇域发展生机活力。落实市直部门联系包保乡镇责任，在工业发展、招商引资和项目建设上实现新突破。抓好乡镇财源培植，力争公共财政预算收入过亿元乡镇达到36个。

　　（六）以改革开放为动力，增创发展新优势。坚持用改革的办法破解难题，用开放的思路拓展空间，为经济社会发展增添活力。

　　深化重点领域、关键环节的改革。稳妥推进事业单位分类改革。加快财税体制改革，健全县级基本财力保障机制，增强基层政府保障能力。加强预算管理，提高预算执行的均衡性、有效性和刚性约束。做好营业税改征增值税试点准备工作，加强政府性债务的清理、监管和风险防范。改革投融资体制，规范发展投融资平台，健全政府投融资、担保平台决策、监督、执行机制，增强调控和配置社会资本的能力，推进资源资本化进程。加快国有企业股权多元化改革，全面推行外派监事会和外部董事制度，完善法人治理结构，加强企业国有资产集中统一监管。深化卫生体制改革，稳妥推进县级公立医院综合改革试点，探索运用市场化手段建设医疗机构基础设施。

　　提升对外开放水平。实施更加积极主动的开放战略，构筑全方位、宽领域、多层次对外开放格局。调整优化出口产品结构，强化工程机械、机电、大蒜等出口基地建设，扩大先进制造业特别是高新技术产品出口比重。加快建设济宁保税物流中心，改进通关模式，提高通关效率。推进“境外百展市场开拓计划”，巩固传统市场，开拓新兴市场，增加市场占有份额。积极扩大进口，鼓励企业大力引进国际高端产业、技术装备和重要原材料。引导企业积极“走出去”，开展境外资源开发、优势产能转移和境外并购，培育一批源自济宁的跨国公司。

　　（七）以实施创新驱动战略为抓手，提升自主创新能力。全面落实科技人才优先发展、优先投入的政策举措，推动国家创新型试点城市建设取得突破，增强转型发展的驱动力。一是突出企业创新主体地位。实施企业技术创新工程，引导企业持续加大研发投入，强化技术攻关、新品开发，提升核心竞争力，重点企业研发经费支出占比达到5%以上，年内新增国家级高新技术企业25家，高新技术产业产值增长20%以上。二是加强产学研合作。深化与高等院校、科研院所的合作，加快校企联盟、产业技术联盟等平台建设，全年新建25家产学研基地、5家省级院士工作站，认定6家省级以上企业技术中心。三是强化公共创新平台建设。放大济宁高新区科技创新功能效应，加强与中关村、张江等高科技园区合作，提高承接高新技术产业和高端人才转移的能力。提升鲁南工程技术研究院、鲁南煤化工研究院服务水平与研发能力，省科学院济宁分院、中科院计算所济宁分所年内建成使用。依托鲁南质检中心，组建检测检验公共平台，发展集中统一的食品检测中心。高标准建设科技人才创业创新孵化园，规范发展鲁南技术产权交易中心，促进科技成果加速转化，年内培育重大科技成果160项。加强知识产权保护，优化创新环境。四是促进科技金融融合发展。吸引更多创投基金、产业基金入驻我市，建成科技担保平台，推进知识产权质押和债权性、股权性融资业务，形成多元化、多层次、多渠道的科技投融资体系。五是抓好人才队伍建设。深入实施海外人才引进“511”计划和国内人才集聚“百千万”工程，引进一批创新团队和高端人才带资金项目到我市创业，培养更多技能型、实用型人才。大力倡树尊重人才、尊重创新的浓厚氛围，努力把济宁打造成创新之城、活力之城。

　　（八）以生态环境治理为重点，努力建设“美丽济宁”。实施 “生态突破”战略，着力推进绿色发展、循环发展、低碳发展，努力打造天蓝地绿、山清水秀、生态宜居的美好家园。

　　扎实推进节能减排。严格落实目标责任，实行合同能源管理和能耗总量、项目单耗双重目标控制，严禁新上高耗能、高排放项目。大力发展循环经济，全面推行清洁生产，健全落后产能退出机制，完成70家企业落后产能淘汰任务，构建生态型产业体系，建设一批生态型产业园区。加强大气污染综合治理，抓好脱硫、脱硝高效除尘改造、扬尘污染防治。完善“治用保”防控体系，加快南水北调治污工程建设，强化污水处理厂运营监管，严厉打击违法排污行为，确保水质稳定达标，实现一泓清水安全过境。

　　强化生态环境保护。积极创建全国“绿化模范城市”和全省“湖泊生态修复示范市”，实施10项绿化会战重点工程，新增造林20万亩。推进环南四湖大生态带建设，完成湿地保护修复20万亩。建设绿色生态廊道，保护林地、湿地和城市公园等绿色空间。严守耕地红线，节约集约用地，严查违法用地和违规采矿行为，治理采煤塌陷地1.5万亩。开展生态示范创建，扩大生态县乡村覆盖面，支持曲阜、泗水争创国家级环保模范城市，新增省级绿化模范县、森林城市、园林城市3个，绿化示范村100个。

　　深化农村环境综合治理。以农村道路硬化、环境净化、村庄美化为重点，继续实施“村村通、村内通”工程，完成1700个村庄、5100公里道路硬化。搞好农村垃圾集中处置，构建城乡环卫一体化管理长效机制，从根本上改变农村环境面貌。

　　（九）以构筑文化高地为目标，推进文化强市建设。加强社会主义核心价值体系建设，提升文化软实力，加快建成文化强市。

　　强力实施文化建设“突破曲阜”战略。抓住文化部与山东省共建曲阜文化经济融合发展创新示范区机遇，完善曲阜文化建设规划，落实扶持政策，争取国家支持，倾力打造文化经济特区。加快曲阜国家级文化产业示范园区建设，优化园区布局，理顺运行体制，引进一批文化项目。加紧推进孔子博物馆等重大项目建设。高水平办好国际孔子文化节和世界儒学大会，提升祭孔大典层次水平，打响孔子品牌。

　　提升文化产业竞争力。加强文化产业集聚区建设，促进文化产业与科技、旅游、金融深度融合，提高文化产业在经济总量中的比重。加快东方文博城等项目建设进度，做好大运河济宁段保护申遗工作。搞好文化资源市场化运作，积极推进与国内知名文化企业的合作。支持有条件的文化企业跨行业兼并重组，培育发展一批传媒、演艺、出版等大型文化企业集团。

　　繁荣发展文化事业。深入开展群众文化活动，大力实施文化惠民工程，办好市民大舞台、百姓大舞台，丰富群众精神文化生活。年内完成市杂技城主体工程和图书馆新馆规划设计。巩固扩大文艺院团改革成果，壮大文化人才队伍，创作一批文艺精品，精心编排第十届中国艺术节参演剧目。

　　扎实做好第23届省运会、第9届省残运会和第3届省老年人运动会各项筹备工作，搞好场馆建设、备战参赛、市场运营和服务保障，营造“我为省运添光彩”浓厚氛围，确保所有场馆全部建成通过验收。广泛开展全民健身活动，推动竞技体育和群众体育、体育事业和体育产业协调发展。

　　（十）以增进百姓福祉为落脚点，加强以保障改善民生为重点的社会建设。持续加大民生投入和政策扶持，健全民生保障十大体系，推动公共服务资源向基层延伸。实施城乡居民收入倍增计划，继续办好为民承诺10件实事。

　　优先发展教育，加快实现“学有优教”。推进高校与地方融合发展、扩大规模、提高水平。全面落实职业培训普惠制政策，推动职业教育集团化办学。加快县市区中小学办学条件标准化建设，完成学前教育三年行动计划和校舍安全工程。调整优化中小学、幼儿园规划布局，逐步解决教育资源不足、布局不合理问题。鼓励引导社会力量兴办教育。关爱中小学生健康成长，扩大营养改善覆盖范围，建设完善400所农村学校食堂，提高家庭经济困难学生资助水平，让每一个孩子都能够享受到更好教育！

　　努力扩大就业，加快实现“劳有多得”。扎实做好以高校毕业生为重点的青年就业，统筹抓好农村转移劳动力、城镇困难人员、退役军人就业工作。持续加大促进就业创业政策力度，大力推动自主创业。支持劳动密集型企业发展，创造更多就业岗位。年内新增城镇就业7万人，农村劳动力转移就业7万人，高校毕业生就业率达到85%，“零就业”家庭实现动态消零。通过扩大就业、支持创业、鼓励投资等措施，多渠道增加城乡居民收入，让每一个劳动者生活得更有尊严！

　　健全社保体系，加快实现“老有善养”。强化社会保险扩面征缴，构建起全覆盖、保基本、多层次、可持续的社会保障体系。加大济困救助力度，提高社会救助标准，大力发展社会慈善事业，落实孤儿基本生活保障制度。健全残疾人保障服务体系。全市城乡居民养老保险基础养老金平均65元以上。完善养老服务体系，12个县市区全部建成社会福利中心，推进乡镇敬老院、社区和村级互助养老服务设施建设，提高社会养老服务水平，让每一个老年人都能幸福安享晚年！

　　优化医疗服务，加快实现“病有良医”。提高城镇职工、城镇居民基本医疗保险统筹基金和大额医疗救助金支付限额，城镇职工提高到每人每年25万元、城镇居民提高到13万元，新农合筹资标准提高到350元以上，重大疾病报销限额达到20万元。完善三级医院“先看病后付费”配套措施，全面推广“病历费用双公开”。做好4万名孕期妇女产前筛查和无创基因检测。推行农村保健医生制度，搞好基层医疗人员培训，开展“万名医生进农户”活动，把最优质的医疗服务送到群众家门口！

　　改善住房条件，加快实现“住有宜居”。严格执行国家房地产宏观调控政策，促进房地产市场健康发展。多渠道解决群众住房困难问题，新建经济适用房5250套、公共租赁房3150套、廉租房950套，改造棚户区2716户、农村危房1.2万户，启动农村住房集中建设4万户，让人民群众安居乐业、生活更加美满！

　　加强和创新社会管理。强化基层基础工作，完善社区（村）管理体制和服务网络，健全综合服务平台，提升服务管理功能。加强社会组织的培育和管理，更好地发挥群众性组织的作用。加强社会矛盾隐患排查，推进社会稳定风险评估工作，健全矛盾纠纷专业调处机制，从源头上预防和减少社会矛盾。深入开展领导干部接访下访活动，扩大12345市长公开电话覆盖范围，畅通民意诉求渠道。加强和完善网络信息管理，营造良好网络环境。深化“平安济宁”建设，构建社会治安网格化防控体系，严厉打击各类违法犯罪活动。严格落实安全生产“一岗双责”，深入开展交通、建筑、矿山安全等专项整治，加强应急管理，有效防范和坚决遏制重特大安全事故发生。强化价格调节基金征缴使用，保持价格总水平基本稳定。加大食品安全和粮食监管执法力度，确保人民群众饮食用药安全。深入开展双拥共建活动，加强国防动员、人民防空、民兵预备役建设，落实各项优待安置政策，圆满完成征兵任务。切实保障妇女儿童权益。稳定低生育水平，改善出生人口性别比。扎实推进对口援疆工作。统筹做好民族宗教、外事侨务和对台工作，提升统计、水文、防汛、气象、史志和防震减灾、住房公积金等项工作水平。

　　三、加强政府自身建设

　　认真贯彻中央和省、市委关于改进工作作风、密切联系群众的各项规定，坚持以人为本、执政为民，加强作风建设，转变政府职能，努力建设人民满意的学习型、服务型、创新型政府。

　　一是加强学习，提升发展境界。深入学习党的十八大精神，努力把党的最新理论成果转化为谋划科学发展的思路办法、领导科学发展的实际能力、促进科学发展的具体措施。完善知识结构，更加注重学习经济、科技、金融、法律法规和现代管理知识，增强分析研判和解决实际问题的能力。强化改革意识、开放意识和市场意识，以创新的理念和方法寻求新的突破。

　　二是转变职能，提高服务效率。深化行政审批制度改革，进一步清理和精简行政审批事项，减少政府对微观经济活动的直接干预。加大政务公开力度，加强电子政务建设，做好“微博问政”，健全咨询、问责制度，让权力在阳光下运行。严格落实岗位责任制、首问负责制、按时办结制，坚决查处“三乱”行为，大力提高办事效率和工作水平。

　　三是改进作风，强化工作落实。各级政府和领导干部要崇尚实干，始终保持担当的精神、创业的激情、进取的锐气，始终做到事业为先、责任为重。坚决扑下身子，深入建设一线、项目现场，努力把问题解决在一线、任务完成在一线。要切实改进调查研究，多到困难矛盾多、群众意见大的地方研究问题、解决困难、指导工作。下基层要轻车简从、减少陪同、简化接待，不张贴标语、不悬挂横幅。进一步改进会风文风，大力精简会议文件，严格清理、大幅减少会议活动，控制会议规模和时间，提倡开短会、讲短话、戒空话。

　　四是依法行政，规范政务行为。自觉接受市人大及其常委会的监督，认真落实市人大及其常委会的各项决议、决定，并定期报告工作。积极支持人民政协履行政治协商、民主监督、参政议政职能，认真听取各民主党派、工商联、无党派人士和各人民团体的意见建议。及时办理人大代表建议和政协提案。深入实施依法行政“五五”规划。加强和完善行政程序制度，确保政府决策科学民主、依法合规。规范行政执法行为，深化行政复议改革，完善行政争议解决机制。

　　五是廉洁自律，树立良好形象。注重加强党性和道德修养，严格遵守廉洁从政各项规定，全面落实党风廉政建设责任制，强化对领导班子和领导干部的教育、管理和监督。进一步规范行政权力，对政府投资的重大项目、政府采购、土地出让等经济行为，严格按制度和程序进行。严格执行住房、车辆配备等有关规定，坚决反对铺张浪费。今年市本级行政经费实现“零增长”，切实把有限的资金和财力用在促发展、保民生上。建立完善惩治和预防腐败体系，强化审计监督，支持纪检监察机关依法查办案件，严厉惩治腐败行为，以为民、务实、清廉的良好形象赢得人民群众的信任和支持。

　　各位代表！新的征程已经开启，新的目标催人奋进。让我们以深入贯彻落实党的十八大精神为动力，在中共济宁市委的正确领导下，以更加昂扬向上的精神迎接挑战，以更加奋发有为的斗志开拓进取，以更加求真务实的作风扎实工作，为全面建成小康社会而努力奋斗！
